# Supplementary material for: Locally collective hydrogen bonding isolates lead octahedra for white emission improvement
Source: Nat Commun. 2019 Nov 15;10:5190. doi: 10.1038/s41467-019-13264-5 (PMC6858351; doi:10.1038/s41467-019-13264-5)
Supplement: Supplementary file 1 — Supplementary Information [file 41467_2019_13264_MOESM1_ESM.pdf]

Supporting Information for:

Locally collective hydrogen bonding isolates lead octahedra for white-emission improvement

Bin-Bin Cui[1,2], Ying Han[1,2,3], Bolong Huang[4], Yizhou Zhao[3], Xianxin Wu[5,6], Lang Liu[3], Guangyue Cao[1,3], Qin Du[3], Na Liu[3], Wei Zou[7], Mingzi Sun[4], Lin Wang[8], Xinfeng Liu[5], Jianpu Wang[7], Huanping Zhou[9] & Qi Chen[1,3]

---

[1] Advanced Research Institute of Multidisciplinary Science, Beijing Institute of Technology (BIT), Beijing 100081, P. R. China. [2] School of Chemistry and Chemical Engineering, BIT, Beijing 102488, P. R. China. [3] Beijing Key Laboratory of Construction Tailorable Advanced Functional Materials and Green Applications, Experimental Center for Advanced Materials, School of Materials Science and Engineering, BIT, Beijing 100081, P. R. China. [4] Department of Applied Biology and Chemical Technology, The Hong Kong Polytechnic University Hung Hom, Kowloon, Hong Kong, P. R. China. [5] CAS Key Laboratory of Standardization and Measurement for Nanotechnology, CAS Center for Excellence in Nanoscience, National Center for Nanoscience and Technology, Beijing 100190, China.[6] University of Chinese Academy of Sciences, Beijing 100049, China. [7] Key Laboratory of Flexible Electronics & Institute of Advanced Materials, Jiangsu National Synergetic Innovation Center for Advanced Materials, Nanjing Tech University, Nanjing 211816, P. R. China. [8] School of Mechatronical Engineering, BIT, Beijing 102488, P. R. China. [9] Department of Materials Science and Engineering, College of Engineering, Peking University, Beijing 100871, P. R. China. Correspondence and requests for materials should be addressed to B.B.C. (email: cui-chem@bit.edu.cn), B.H. (email: bhuang@polyu.edu.hk) and Q.C. (email: qic@bit.edu.cn).

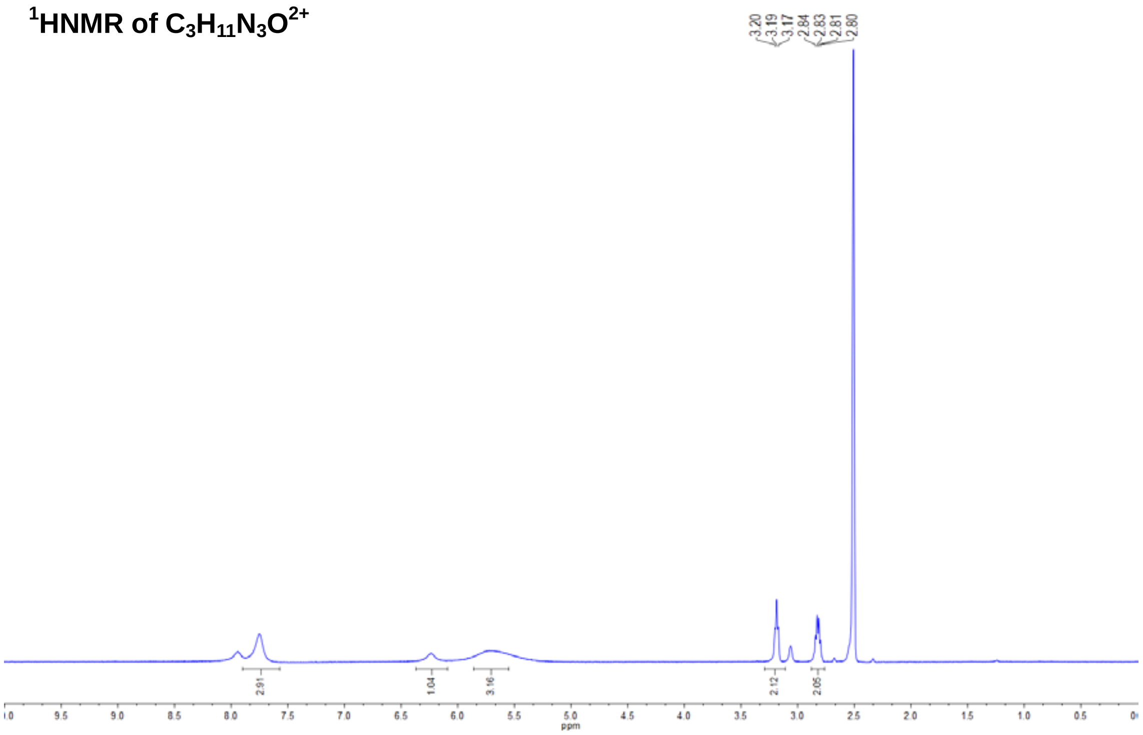

Supplementary Figure 1 | ^{1}HNMR spectrum of $C_3H_{11}N_3O^{2+}$ in DMSO-d6.

^{1}H NMR (DMSO-d6): δ 2.80 (q, J = 6.0 Hz, 2 H, $CH_2NH_3^+$), 3.20 (t, J = 6.0 Hz, 2 H, $CONHCH_2$), 5.72 (s, 3 H, $CONH_3^+$), 6.22 (s, 1 H, $CONHCH_2$), 7.71 (s, 3 H, $^+NH_3$).

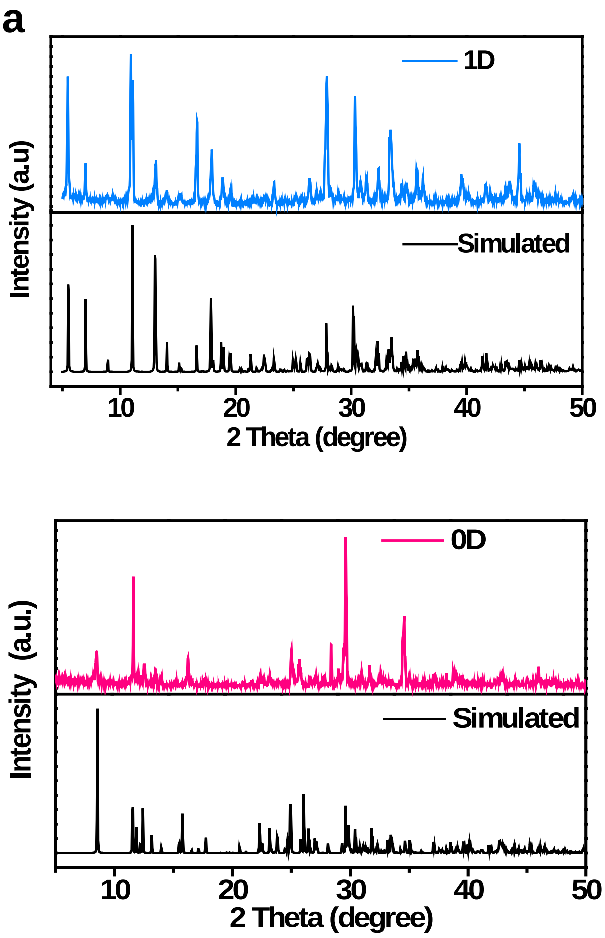

Supplementary Figure 2 | Powder X-ray diffraction (PXRD) spectrum of 1D (a) and 0D (b) bulk crystals, respectively, as well as the single crystals based simulated results.

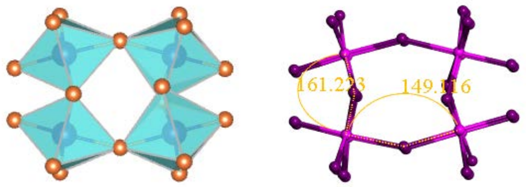

Supplementary Figure 3 | View of an individual 1D lead bromide quantum-wire with corner sharing octahedra along the c-axis.

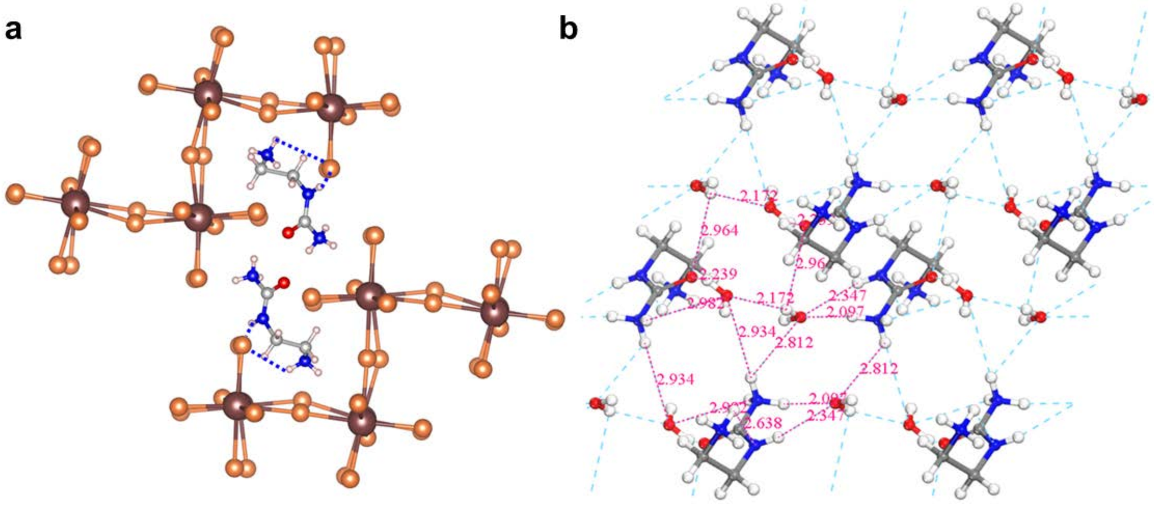

Supplementary Figure 4 | (a) Local hydrogen bonding causes the unique bending of the 1D structure. (b) View of hydrogen-bonding in organic cations and water of the bulk 0D lead bromide bulk crystal.

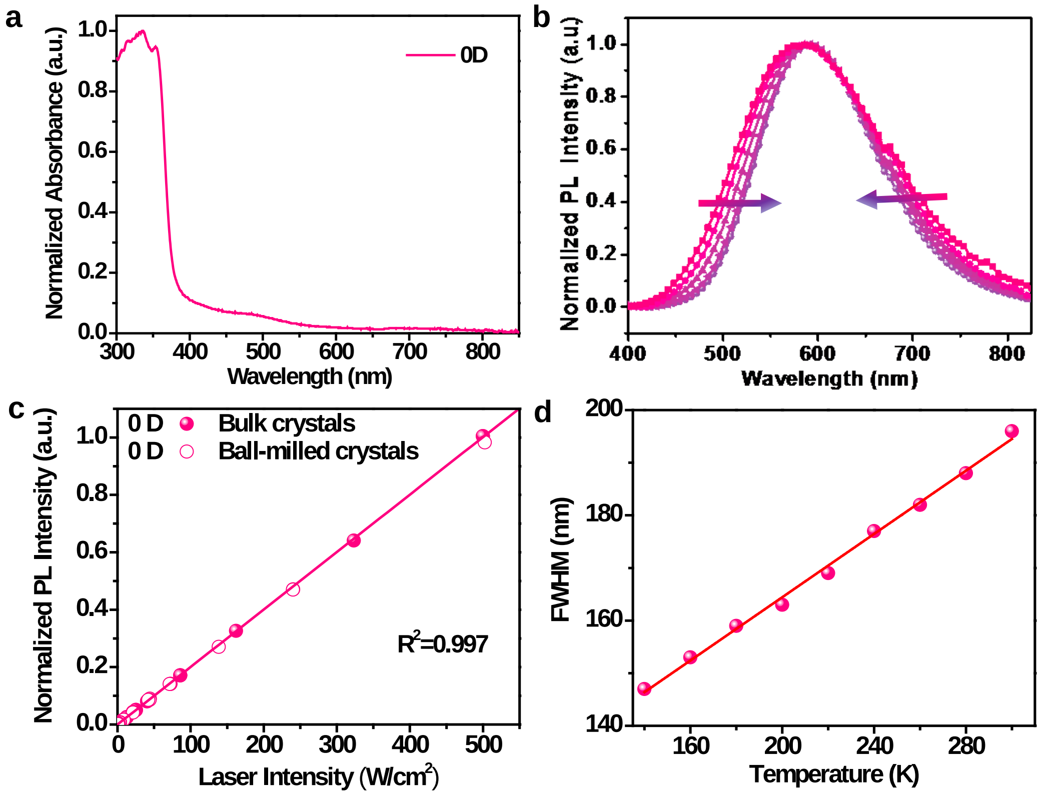

Supplementary Figure 5 | (a) Optical absorption spectra of the 0D bulk crystal. (b) Temperature dependence of the emission from the 0D crystal. (c) PL intensity versus excitation power for the 0D bulk crystal at room temperature. (d) Full width at half-maximum (FWHM) of the 0D crystal follows a linear trend with increasing temperature.

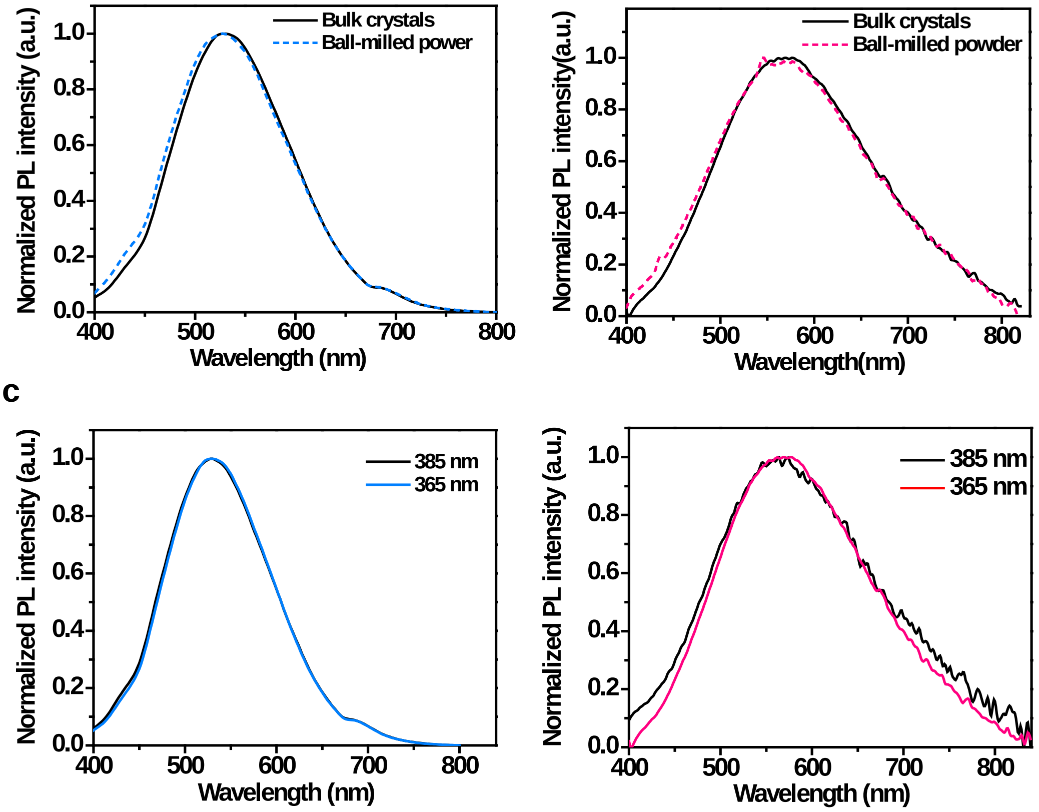

Supplementary Figure 6 | Emission spectra of 1D bulk and ball-milled crystals (a) and 0D bulk and ball-milled crystals (b) under 365 nm excitation. Emission spectra of 1D (c) and 0D (d) bulk crystals at different excitation wavelengths.

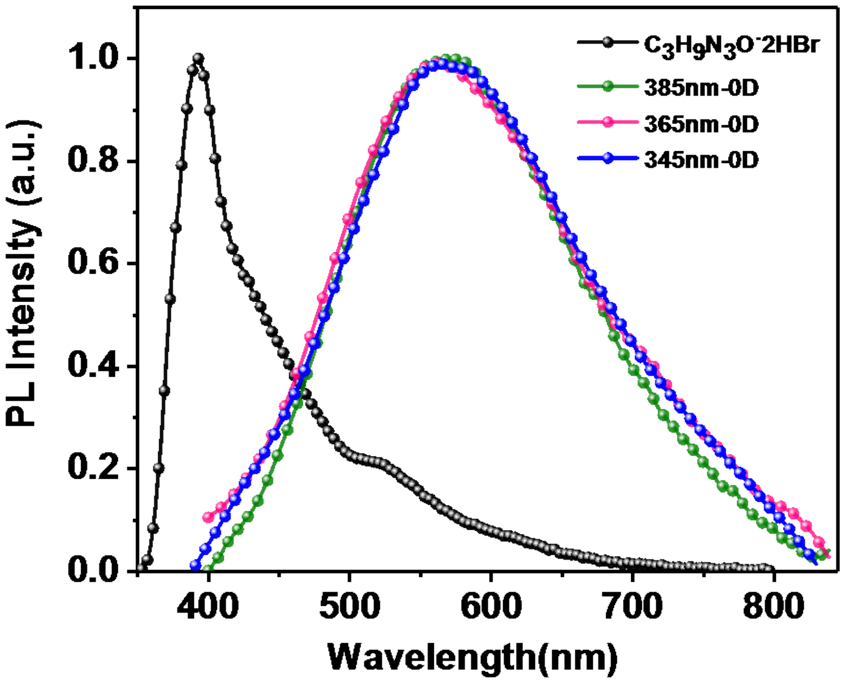

Supplementary Figure 7 | PL of 0D single crystals are independent on different excitation wavelengths and the PL of organic salts.

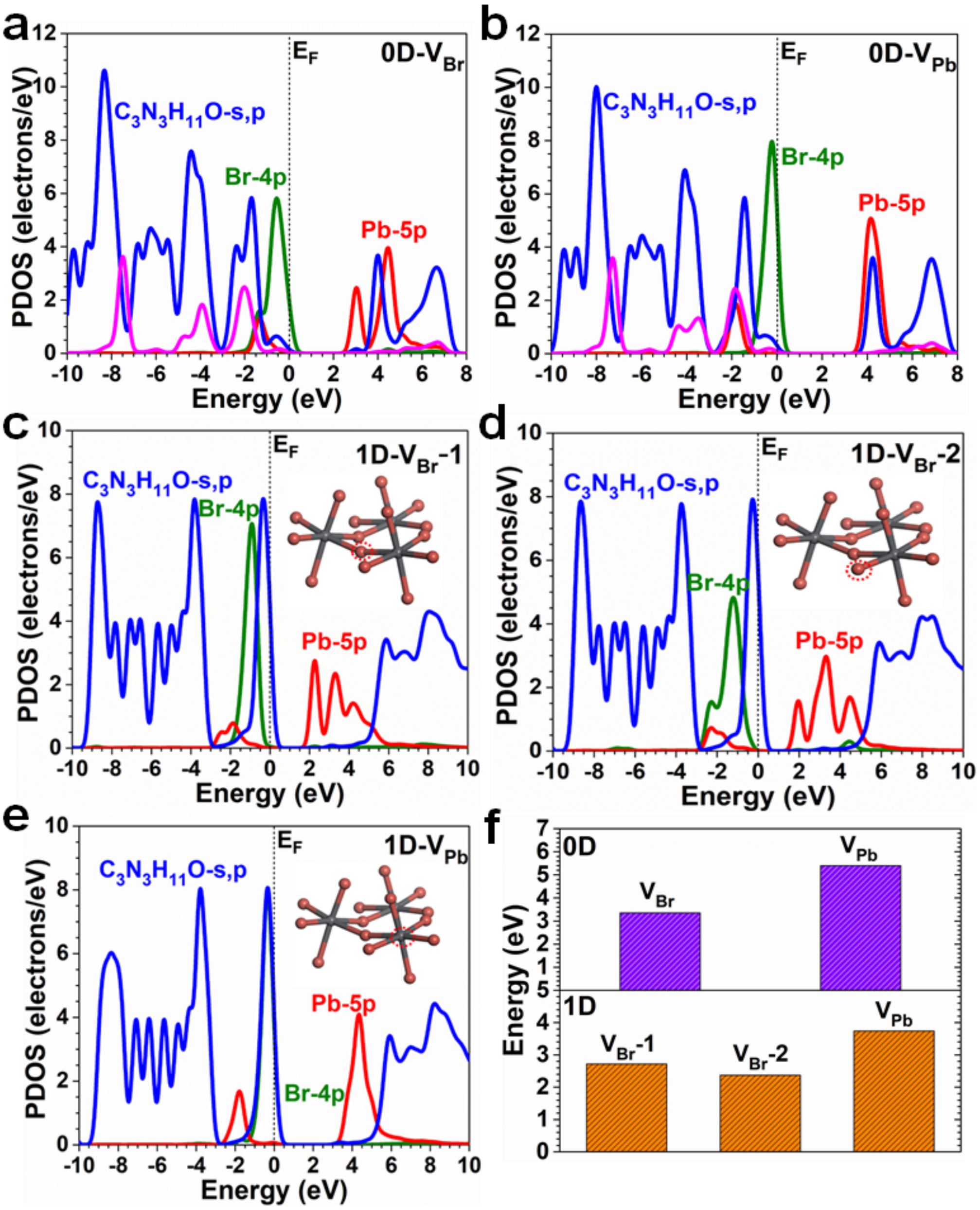

Supplementary Figure 8 | Density functional theory (DFT) calculations on intrinsic defects. (a, b) PDOS of Br and Pb vacancies in 0D lead bromide hybrids. (c-e) PDOS of Br and Pb vacancies in 0D lead bromide hybrids. (f) Calculated formation energies of vacancies in 0D and 1D lead bromide hybrids.

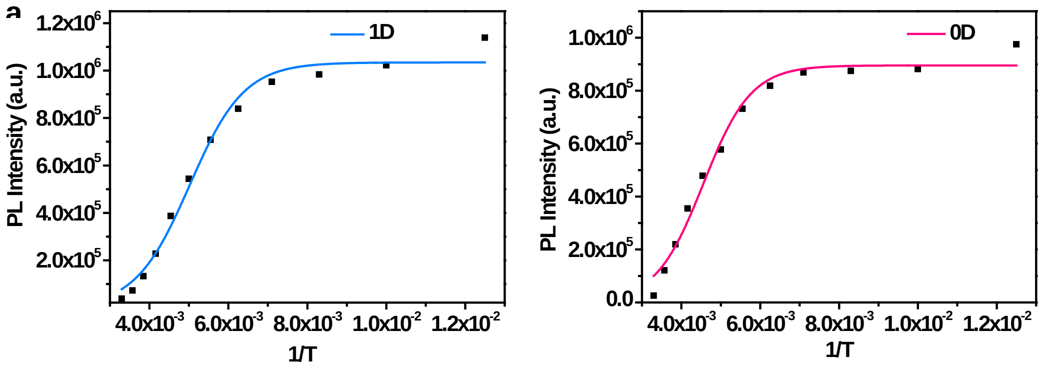

Supplementary Figure 9 | The integrated PL emission intensity with respect to 1/T. Exciton binding energy was estimated using the following fitting:

$$I_T = I_0 / [1 + A\exp(-E_B/k_BT)]$$

Where I_T is the integrated intensity at T K, E_B is the binding energy, and k_B is the Boltzmann constant. where I_0 is the estimated integrated PL intensity at 0 K.

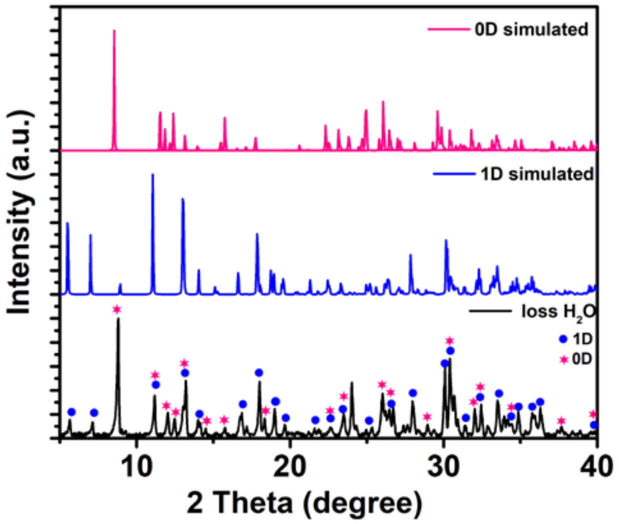

Supplementary Figure 10 | Powder X-ray diffraction (PXRD) pattern of bulk 0D crystals after loss H_2O, as well as the simulated PXRD patterns of 0D and 1D single crystal structure.

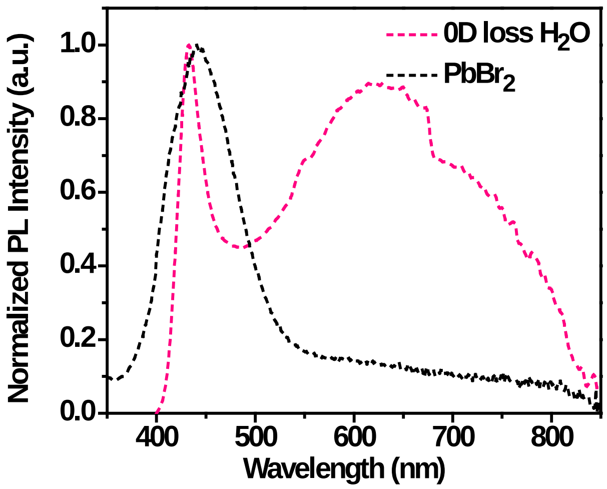

Supplementary Figure 11 | Emission spectra of bulk 0D crystals after loss H_2O.

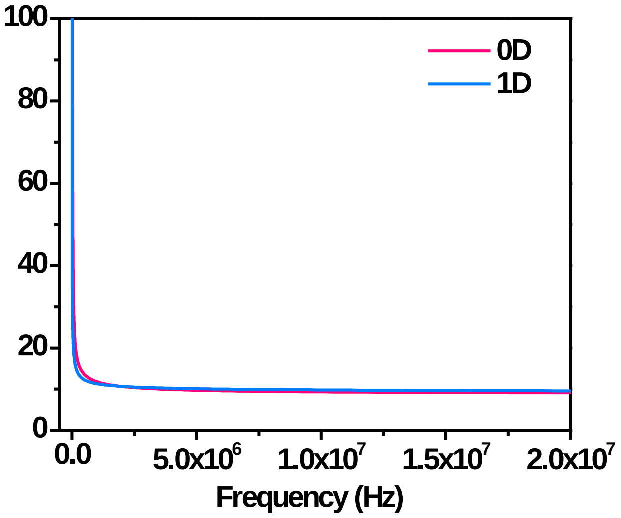

Supplementary Figure 12 | Dielectric constant, ε' versus frequencies measured on 0D and 1D materials.

Supplementary Table 1 | Single crystal X-ray diffraction data of 1D and 0D bulk single crystals.

Identification code	1D	0D
Empirical formula	$C_9H_{32}Br_9N_9O_3Pb_2$	$C_6H_{30}Br_6N_6O_6Pb$
CCDC No.	1894525	1894526
Formula weight	1448.00	969.01
Temperature/K	153.15	153.15
Crystal system	monoclinic	triclinic
Space group	$P2_1/c$	P-1
a/Å	16.486(3)	8.1354(16)
b/Å	25.216(5)	8.1690(16)
c/Å	8.3385(17)	10.604(2)
α/°	90	105.79(3)
β/°	103.97(3)	90.17(3)
γ/°	90	114.29(3)
Volume/$Å^3$	3363.9(12)	612.8(3)
Z	4	1
Density (calcd)/$g \cdot cm^{-3}$	2.859	2.626
Absorption coefficient/mm^{-1}	20.714	16.695
F(000)	2608.0	448.0
Crystal size/mm^3	0.13 × 0.1 × 0.05	0.13 × 0.12 × 0.1
Radiation	MoKα (λ = 0.71073)	MoKα (λ = 0.71073)
2Θ range for data collection/°	3.014 to 54.84	5.542 to 54.988
Index ranges	$-20 \leq h \leq 21$, $-32 \leq k \leq 31$, $-10 \leq l \leq 10$	$-9 \leq h \leq 10$, $-10 \leq k \leq 10$, $-13 \leq l \leq 13$
Reflections collected	22180	5671
Independent reflections	7596 [R_{int} = 0.0943, R_{sigma} = 0.0990]	2788 [R_{int} = 0.0668, R_{sigma} = 0.0614]
Data/restraints/parameters	7578/354/329	2788/0/115
Goodness-of-fit on F^2	1.100	1.139

Final R indexes [I>=2σ (I)]	R_1 = 0.1398, wR_2 = 0.2857	R_1 = 0.0480, wR_2 = 0.1215
Final R indexes [all data]	R_1 = 0.1545, wR_2 = 0.2943	R_1 = 0.0498, wR_2 = 0.1231
Largest diff. peak/hole/e Å^{-3}	3.67/-7.11	2.11/-3.73

Supplementary Table 2 | Hydrogen Bond Strength for 0D lead bromide hybrids

Structure	0 D
Number of Hydrogen bonds	8
Total Hydrogen-Bonding Energy (eV)	2.13
Hydrogen-Bonding Energy (eV)	0.27

Supplementary Table 3 | Fractional Atomic Coordinates (×10^4) and Equivalent Isotropic Displacement Parameters (Å^2×10^3) for the 1D bulk single crystal. U_{eq} is defined as 1/3 of of the trace of the orthogonalised U_{IJ} tensor. All atoms were refined with anisotropic displacement parameters.

**Atom**	***x***	***y***	***z***	**U(eq)**
Pb2	3130.4(8)	6614.1(5)	6746(2)	21.4(4)
Pb1	6952.1(7)	6650.7(4)	8530.6(14)	21.6(3)
Br9	1408(2)	6930.8(15)	5379(5)	36.6(8)
Br4	7124(2)	5754.3(13)	6387(4)	30.2(7)
Br7	2744(2)	5917.2(15)	9187(4)	37.8(8)
Br8	2978(2)	5780.9(12)	4174(4)	27.8(7)
Br3	7296(2)	6016.4(18)	11548(5)	32.2(11)
Br2	8715(2)	6943.5(14)	8935(5)	35.3(8)
Br1	6520(3)	7331.9(15)	5292(5)	25.7(11)
Br5	5066(2)	6327.2(17)	8046(5)	43.8(9)
Br6	3431(3)	7511(2)	4369(9)	26.9(17)
O1	9510(14)	5321(9)	3280(30)	27(4)

O3	499(14)	5373(9)	-1260(30)	29(4)
N3	8422(17)	4856(10)	3970(40)	33(5)
N8	1110(18)	5637(11)	1350(40)	31(4)
C7	957(17)	6579(13)	610(40)	33(5)
N9	1577(16)	4881(10)	460(40)	30(5)
N1	8391(16)	6745(11)	4660(40)	33(5)
N2	8977(18)	5658(11)	5350(40)	30(4)
C1	9192(18)	6593(13)	4290(40)	31(5)
C3	9000(19)	5283(12)	4200(40)	29(4)
C2	9520(20)	6105(14)	5610(40)	31(4)
C9	1013(19)	5327(11)	150(40)	29(4)
C8	620(20)	6133(14)	1260(50)	33(5)
N7	1774(16)	6782(12)	1420(40)	37(5)
C5	5140(30)	6081(19)	3590(60)	54(6)
C6	5260(30)	5170(20)	2700(60)	68(6)
N4	5080(20)	6919(14)	1760(50)	54(7)
C4	4630(30)	6499(16)	2370(60)	53(6)
N5	5470(30)	5661(17)	2640(50)	62(6)
N6	5620(30)	4860(17)	1560(50)	69(7)
O2	4500(30)	5021(17)	2350(50)	80(7)
Br1A	6565(12)	7615(8)	6110(20)	41(4)
Br6A	3535(11)	7354(9)	3750(30)	42(4)

Supplementary Table 4 | Hydrogen Atom Coordinates (Å×10^4) and Isotropic Displacement Parameters (Å^2×10^3) for 1D bulk single crystal. All the hydrogens were refined with isotropic displacement parameters.

**Atom**	***x***	***y***	***z***	**U(eq)**
H3A	8232.45	4801.44	2945.97	40
H3B	8021.25	4934.24	4409.17	40
H3C	8664.75	4577.74	4427.17	40
H8	1488.9	5572.28	2215.61	37

H7A	553.95	6873.8	548.05	40
H7B	970.48	6491.09	-541.18	40
H9A	1423.15	4650.92	-300.82	36
H9B	1587.25	4742.32	1388.48	36
H9C	2061.85	4993.42	446.78	36
H1A	8148.24	6987.22	4006.34	40
H1B	8484.94	6854.42	5647.64	40
H1C	8079.74	6471.92	4543.84	40
H2	8633.7	5620.49	5959.2	36
H1D	9590.86	6892.77	4484.35	38
H1E	9108.5	6470.04	3135.85	38
H2A	10082.9	5990.84	5502.73	37
H2B	9583.58	6239.05	6746.96	37
H8A	51.82	6067.03	581.19	39
H8B	573.55	6221.84	2393.34	39
H7C	2160.35	6628.94	1080.06	44
H7D	1834.25	6717.94	2442.86	44
H7E	1800.65	7114.84	1275.06	44
H5A	5604.11	6257.5	4379.43	65
H5B	4769.21	5915.16	4231.48	65
H4A	4742.74	7098.83	1020.11	65
H4B	5445.54	6776.03	1346.81	65
H4C	5309.64	7122.33	2548.71	65
H4D	4300.19	6304.36	1394.76	63
H4E	4225.42	6667.87	2913.1	63
H5	5763.61	5758.79	1978.2	75
H6A	6134.24	4931.39	1660.78	82
H6B	5562.14	4527.49	1660.78	82

Supplementary Table 5 | Fractional Atomic Coordinates ($\times10^4$) and Equivalent Isotropic Displacement Parameters ($Å^2\times10^3$) for the 0D bulk single crystal. U_{eq} is

defined as 1/3 of of the trace of the orthogonalised U_{IJ} tensor. All atoms were refined with anisotropic displacement parameters.

**Atom**	***x***	***y***	***z***	**U(eq)**
Pb1	5000	0	5000	8.90(14)
Br2	5887.4(9)	2520.4(10)	3259.5(7)	13.06(18)
Br3	1125.8(9)	-1607.6(10)	3740.2(8)	16.52(19)
Br1	4649.2(10)	3011.1(11)	7224.4(7)	17.59(19)
O3	8095(8)	5204(8)	1220(5)	19.0(12)
O1	8767(8)	6804(8)	9425(5)	15.4(11)
O2	6907(9)	1468(9)	9321(6)	28.3(14)
N3	6843(9)	8240(10)	9968(6)	18.1(13)
C3	8102(10)	7952(11)	9276(7)	14.3(14)
N2	8730(9)	8796(9)	8361(6)	12.4(12)
N1	7735(9)	6732(9)	5506(6)	15.4(12)
C1	9048(10)	6597(10)	6385(7)	14.2(14)
C2	10010(9)	8391(10)	7512(7)	13.1(14)

Supplementary Table 6 | Hydrogen Atom Coordinates (Å×10^4) and Isotropic Displacement Parameters (Å^2×10^3) for 0D bulk single crystal. All the hydrogens were refined with isotropic displacement parameters.

**Atom**	***x***	***y***	***z***	**U(eq)**
H3D	8829	6346	1409	29
H3E	7419	5042	1814	29
H2C	6719	2024	8815	42
H2D	7330	2304	10054	42
H3A	5749	7482	9509	22
H3B	7026	9433	10123	22
H3C	6904	8003	10734	22
H2	8370	9617	8275	15

H1A	7196	5649	4861	19
H1B	8305	7662	5158	19
H1C	6901	6958	5971	19
H1D	9962	6321	5866	17
H1E	8402	5541	6750	17
H2A	10932	8269	8043	16
H2B	10647	9453	7151	1

Supplementary Table 7 | Parameter comparison between our 0D and reported Cs_4PbBr_6.

	dielectric constant	exciton binding energy (meV)
0D (this work)	11	141
Cs_4PbBr_6	7.7	159 ± 18
$(C_4N_2H_{14}Br)_4SnBr_6$	11.9	N / A
$(C_4N_2H_{14}I)_4SnI_6$	7.7	N / A

Supplementary Note 1

For the trap states, we have supplied a detailed discussion. In 0D and 1D systems, different electronic structures are noticed for vacancy states. In 0D, the Br-4p bands localized below the EF while the anti-bonding orbitals of Pb-6p occupy 1 eV below the CB (Supplementary Figure S8a). Similarly, the Br-4p band is still pinned near the EF in defective 0D system with Pb vacancy. However, the strong coupling between Pb-6p and organic spacer induces the elimination of anti-bonding states of Pb (Supplementary Figure S8b). In contrast, the organic spacer occupies a higher position than that of the Br-4p bands in different Br vacancy models. This indicates that the Br vacancy only contributes to the hole distribution near anti-bonding of Pb rather than involving into the modulation of trap states (Supplementary Figure S8c-S8d). Interestingly, we notice the well-matching bands between Br and organic spacer, which leads to the absence of hole states below CB (Supplementary Figure S8e). Finally, we compare the formation of vacancies in both 0D and 1D systems. Apparently, Br vacancies show smaller energy cost than Pb vacancies due to the coordination environment. The connected Br atom in [Pb_2Br_9] unit also shows higher energy barrier in the formation than the normal Br atoms. The formation of Br vacancies is in the range of the excitation energy, supporting the potential contribution of Br vacancy (Supplementary Figure S8f).
